# Characterization of the complete mitochondrial genome of *Ostorhinchus novemfasciatus* and phylogenetic studies of Apogoninae

**DOI:** 10.1080/23802359.2020.1845579

**Published:** 2021-01-05

**Authors:** Ying Sun

**Affiliations:** Marine Science and Technology College, Zhejiang Ocean University, Zhoushan, China

**Keywords:** *Ostorhinchus novemfasciatus*, mitochondrial genome, phylogenetic analysis

## Abstract

The complete mitochondrial genome of *O*storhinchus *novemfasciatus* was first presented in this study. The full length of the mitochondrial genome was 16,779 bp, including 13 protein-coding genes (PCGs), two ribosomal RNAs, 22 transfer RNA genes, a non-coding control region (CR) and one origin of replication on the light-strand (OL). The total nucleotide composition of mitochondrial DNA was 26.0% A, 30.2% C, 26.2% T and 17.5% G. 12 PCGs used the canonical ATG as their initiation codon, whereas COI gene started with an alternative start codon GTG. The mitochondrial genome of *O. novemfasciatus* described in this study could be a useful basis for the management of this species and laid a foundation for further research involved with phylogenetic relationship within Apogoninae.

*O*storhinchus *novemfasciatus* widely distributed in the eastern Indian Ocean, it was common in shallow lagoons, as well as rocky reefs (Yoshida et al. 2010). As a small economic fish in Zhoushan Islands, *O. novemfasciatus* is often purchased for aquaculture feed processing (Shao et al. 2007). Besides, the genus *Ostorhinchus* was composed of nearly 60 species, however, the genetic and molecular information of *Ostorhinchus* is limited (Zhu et al. 2019). In this study, we described the complete mitochondrial genome of *O. novemfasciatus* and explored its phylogenetic position within Apogoninae, to gain its molecular information which was expected to contribute to purchasing management of *O. novemfasciatus*, as well as further phylogenetic studies on its related species.

An individual specimen of *O. novemfasciatus* was collected from the Jiulong River (Fujian province, China, N24°58′63.99″, E118°16′86.73″) and stored in the Research Center of Zhejiang Ocean University with accession number 20200916SY03. The total genomic DNA was extracted from a portion of the epaxial musculature using the phenol–chloroform method (Barnett and Larson 2012). The complete mitogenome of *O. novemfasciatus* was amplified with the help of 16 pairs of primers, that were designed based on universal primers for amplification of mitogenomes in marine fish species (Cheng et al. 2012; Shao et al. 2014). Fragments generated from PCR amplification were sequenced using Sanger sequencing technology. Sequenced fragments were assembled to create the complete mitogenome using CodonCode Aligner 5.1.5 (CodonCode Corporation, Dedham, MA). The complete mitogenome was annotated using the software of Sequin (version 15.10, http://www.ncbi.nlm.nih.gov/Sequin/). Transfer RNA genes and their potential cloverleaf structures were identified using tRNAscan-SE 1.21 (Lowe and Eddy 1997).

The complete mitogenome of *O. novemfasciatus* was 16,779 bp in length (GenBank accession number MW007385), containing 13 protein-coding genes (PCGs), 2 ribosomal RNA genes (12S and 16S), 22 transfer RNA (tRNA) genes, one origin of replication on the light-strand (OL) and a putative control region (CR). The overall base composition was 26.0% A, 30.2% C, 26.2% T and 17.5% G, respectively, with a slight AT bias of 52.2%. The gene arrangement, composition and size were quite similar to the teleost fish mitogenomes published previously (Jing et al. 2010; Zhu et al. 2018).

The total length of 13 PCGs of *O. novemfasciatus* mitogenome was 11,430 bp, encoding 3800 amino acid. Similar to the typical vertebrate mitogenome (Miya and Nishida 2000), 12 of them were located on the H-strand, except for *ND6* which was located on the L-strand. All of the PCGs used the canonical ATG initiation codon with the exception of *COI* gene, which started with an alternative start codon GTG. Seven PCGs (*ND2*, *COI*, *ATP8*, *ATP6*, *COIII*, *ND4L*, *ND5*) terminated with the stop codon TAA, two (*ND1*, *ND3*) with TAG, one (*ND6*) with AGG, the genes ended with a single T were *COII*, *ND4* and *Cytb*, the presence of an incomplete stop codon is a common phenomenon in vertebrate mitochondrial genes (Zhu et al. 2018; Lee et al. 2019).

Twenty-two tRNAs dispersed between rRNAs and PCGs were identified by their own anticodon sequences. All the tRNAs were able to fold into a typical cloverleaf structure except for tRNA^Ser (AGC)^ that lacked a dihydrouridine arm (Garey and Wolstenholme 1989). The lengths of the two rRNA genes were 954 bp (12SrRNA) and 1690 bp (16SrRNA) respectively, which located between the tRNA^Phe^ and tRNA^Leu(UUA)^ and interposed by the tRNA^Val^. The control region was detected between tRNA^phe^ and tRNA^Pro^, consisting of 1117 nucleotides, A–T content (61.06%) was higher than G–C content (38.94%).

In order to determine the phylogenetic position of *O. novemfasciatus* within Apogoninae, a phylogenetic tree including seven Apogoninae species was constructed based on the maximum likelihood (ML) method using 13 protein-coding genes, a Kurtidae species (*kurtus gulliveri*) was selected as the outgroup. The ML tree strongly supported that *O. novemfasciatus* was closely related to *Apogon semilineatus* and *Cheilodipterus quinquelineatus* with a bootstrap probability of 99% (Figure 1). Our results were highly in agreement with that of the previous molecular studies (Zhu et al. 2018; Da and Wen 2020).

**Figure 1.**
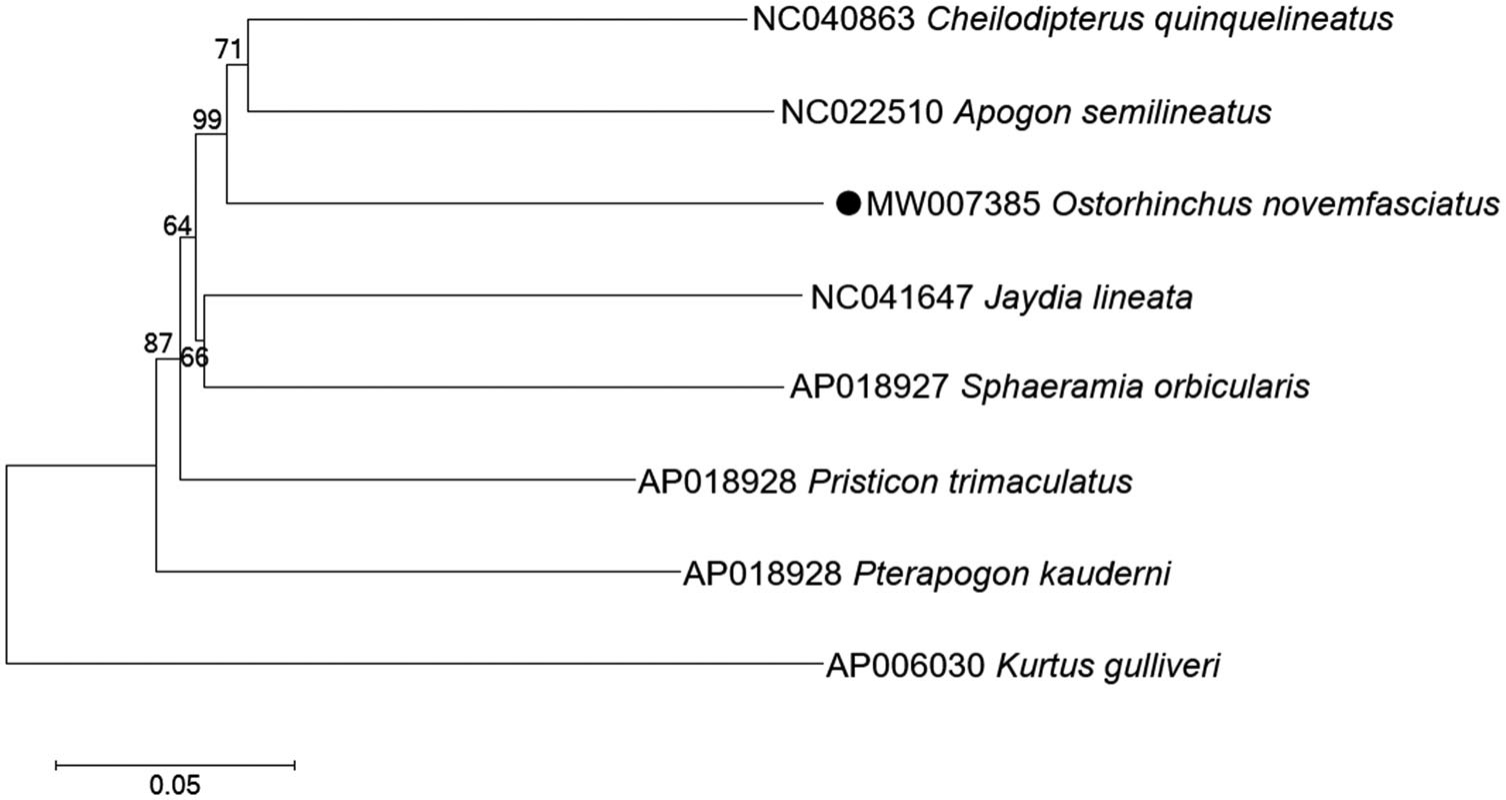
Maximum likelihood (ML) tree of seven Apogoninae species was constructed based on 13 PCGs. The bootstrap values are based on 1000 resamplings. The number at each node is the bootstrap probability. The number before the species name is the GenBank accession number. The mitogenome sequence in this study is labeled with a black dot.

## Data Availability

The data that support the findings of this study are openly available in GenBank of National Center for Biotechnology Information at https://www.ncbi.nlm.nih.gov, reference number MW007385.
